# Frequency and correlates of somatic symptoms in patients of depression: a cross-sectional study at PIMS Islamabad

**DOI:** 10.1097/MS9.0000000000004804

**Published:** 2026-02-23

**Authors:** Fatima Rizwan Bhatti, Remsha Tariq, Rida Hussain, Muddassir Khalid, Sumia Fatima, Laiba Fatima, Qamar Shoukat

**Affiliations:** a Department of Psychiatry, Pakistan Institute of Medical Sciences, Islamabad, Pakistan; b Department of Psychiatry, Nawaz Sharif Medical College, Gujrat, Pakistan; c Department of Medicine, Nishtar Medical University, Multan, Pakistan; d Department of Psychiatry, Rawalpindi Medical University, Rawalpindi, Pakistan

**Keywords:** depression (D003863), diagnosis (D003933), diagnostic and statistical manual of mental disorders (D039721), patient health questionnaire (D000073222), somatic symptoms (D000071896)

## Abstract

**Background:**

Essentially crucial in early diagnosis and treatment of depression are the somatic symptoms, which can significantly decrease the morbidity and mortality associated with these symptoms and depression, as well as decrease the costs of investigation and treatment for the primary healthcare system. We conducted this study to determine the socio-demographic correlates of somatic symptoms in depression in a Pakistani population.

**Methods:**

This cross-sectional study was conducted at the Department of Psychiatry, PIMS Hospital, Islamabad, during the period April 2025 to August 2025. A total of 243 male and female patients aged 12–60 years diagnosed with depression were enrolled. The patients were evaluated for somatic symptoms by conducting an interview based on the PHQ-15 questionnaire. Data was analyzed using SPSS v.26. The primary objective was to determine the prevalence of somatic symptom severity using PHQ-15 categories among patients with DSM-5–diagnosed depression. Secondary objectives were to examine associations between PHQ-15 severity and key sociodemographic predictors including age, gender, education, residence, and marital status.

**Results:**

Gender wise distribution of the patients revealed that 74 males (30.5%) and 169 females (69.5%). In terms of age distribution, the largest age group was 12–25 years (*n* = 131, 53.9%), followed by 26–35 years (*n* = 63, 25.9%). Among the symptoms that bothered the patients a lot, low energy was most frequently reported by 144 patients (59.3%), followed by troubled sleeping by 117 (48.1%). Painful sexual intercourse was least commonly reported by 9 patients (3.7%). High PHQ-15 score was recorded in 52 patients (39.7%), aged 12–25 years, 26 (41.3%) patients aged 26–35 years, 12 (48.0%) patients aged 36–45 years, and 17 (70.8%) patients aged 46–60 years, respectively (*P*-value 0.011).

**Conclusion:**

Most frequently reported somatic symptoms significantly bothering the patients were low energy and trouble sleeping. Age was significantly associated with the degree of somatization, and patients with advanced age were more frequently having a high degree of somatization.

## Introduction

Depressive disorder (also commonly called depression) is one of the most common mental disorders. According to WHO, an estimated 3.8% of the world’s population undergoes depression, including 5% of adults (4% of men and 6% of women)^[1]^. It is characterized by the presence of low mood, lack of interest, or decreased stamina for more than 2 weeks.

**HIGHLIGHTS**

- Somatic symptoms are highly prevalent among depressed patients in Pakistan.
- Fatigue and sleep disturbance were the most frequently reported complaints.
- Severity of somatic symptoms increased significantly with advancing age.
- Gender showed no significant difference in somatic symptom severity.
- Early screening may reduce misdiagnosis and improve timely mental health care.

Somatic symptoms are the physical complaints of patients that are not adequately explained by known pathophysiological phenomena. A comprehensive multicenter WHO study has placed the prevalence of somatic symptoms in depression at 69 percent, with 45–95 percent of depressed patients reporting exclusively somatic symptoms. These findings remained consistent across centers^[2]^.

Studies have shown discrepancies in the expression of somatic symptoms in different cultures. A study in England identified these risk factors for a high score on the Somatic Symptom Inventory: fewer than 12 years of education, separated, widowed, or divorced status, psychological abuse during childhood, co-existing medical illnesses, anxiety, and depression^[3]^.

The most recent study in Pakistan was carried out in 2023 in Khyber Pakhtunkhwa to calculate the prevalence of somatic symptoms in mental illnesses. It found headache (61.9%) and lethargy (41.1%) to be the most commonly experienced somatic symptoms in a psychiatric patient population^[4]^. A 2015 study in Karachi concluded that patients who had medically unexplained symptoms were more educated, had better social support and were financially secure^[5]^.

Studies have shown that somatic presentations pose a significant problem in primary care services by causing diagnostic difficulties. Patients with depression presenting to primary care undergo multiple investigations and are delayed in receiving a psychiatric consultation. Hence, higher rates of somatic presentations significantly contribute to low rates of appropriate recognition in primary care^[6]^.

Higher rates of somatic presentation contribute to increased economic burden on the patient as well as the health system. It heavily contributes to functional impairment for the patient and lowers health-related quality of life. This is especially clinically relevant in a resource-scarce country like Pakistan. This leads to increased burden on primary care services and has important implications for the monetary policy. A 2011 study in Karachi has demonstrated that Pakistani physicians are more likely to investigate underlying physical rather than psychiatric causes of somatic symptoms^[7,8]^.

According to our search on PubMed and Google Scholar, to date, no comprehensive study has been published that investigates the socio-demographic correlates of somatic symptoms in depression in a Pakistani population.

This study had one primary objective:

- to determine the prevalence and distribution of somatic symptom severity using PHQ-15 categories among patients diagnosed with depression in a tertiary-care setting in Pakistan.

The secondary objectives were:

- to evaluate associations between PHQ-15 somatization severity and key sociodemographic variables (age, gender, education, residence, marital status).
- to identify which demographic factors show the strongest statistical relationship with somatic symptom severity.

“This cross-sectional study has been reported in line with the STROCSS guidelines [Agha R, Mathew G, Rashid R, Kerwan A, Al-Jabir A, Sohrabi C, et al. Revised Strengthening the reporting of cohort, cross-sectional and case-control studies in surgery (STROCSS) Guideline: An update for the age of Artificial Intelligence. Premier Journal of Science. 2025]”^[9]^

## Materials and methods

This cross-sectional study was conducted at the Department of Psychiatry at a tertiary care hospital setup in Islamabad, from April 2025 to August 2025. A total of 243 male and female patients aged 12–60 years diagnosed with depression were enrolled. Patients were excluded if they had current psychotic features, bipolar disorder, active substance use disorder, neurological illness, severe or unstable medical conditions (e.g., uncontrolled endocrine, renal, or cardiovascular disease), cognitive impairment affecting interview reliability, or inability to comprehend Urdu/English sufficiently for PHQ-15 administration. After taking approval from the Ethical Review Board of the concerned hospital, patients fulfilling the selection criteria were enrolled from the outdoor department of psychiatry of the hospital.

Sample size was calculated using the standard Cochrane formula for proportions:*n* = *Z*^2^ × *p* (1 – *p*)/*d*^2^

Assuming an expected prevalence (*p*) of 0.30 for somatic symptom severity in depression (based on regional literature), a 95% confidence level (*Z* = 1.96), and a margin of error of 5% (*d* = 0.05), the minimum required sample size was 230 participants.

Informed consent was taken from all enrolled participants after explaining the purpose, risks, and benefits of the study. Non-probability consecutive sampling was employed to select participants who met the inclusion criteria. Depression was diagnosed in patients using DSM-5 criteria. Depression diagnosis was established according to the criteria outlined in the Diagnostic and Statistical Manual of Mental Disorders, Fifth Edition (DSM-5) for Major Depressive Disorder (MDD). A diagnosis required the presence of five or more of the following symptoms during the same 2-week period, representing a change from previous functioning: (1) depressed mood or (2) markedly diminished interest or pleasure in most activities. Additional symptoms included significant weight change or appetite disturbance, insomnia or hypersomnia, psychomotor agitation or retardation, fatigue or loss of energy, feelings of worthlessness or excessive guilt, diminished ability to think or concentrate, and recurrent thoughts of death or suicidal ideation. These symptoms caused clinically significant distress or impairment in social, occupational, or other important functioning domains. The symptoms were not attributable to the physiological effects of a substance or another medical condition. Diagnosis was ascertained through structured clinical interviews conducted by trained clinicians, and inter-rater reliability was regularly evaluated to ensure diagnostic consistency.

Baseline information and demographics like age (years), weight in kg (measured using weighing scale), height in centimeters (measured using stadiometer), BMI (calculated using the formula, weight in kg/height in m^2^), disease (depression) duration (months), residence (rural/urban), education, profession and marital status was recorded. Patients were evaluated for somatic symptoms that they have been experiencing during the course of their illness using the PHQ-15. The Patient Health Questionnaire-15 (PHQ-15) was employed in this study to assess somatic symptom severity due to its established reliability and validity in diverse populations. The PHQ-15 is widely used as a standardized instrument for capturing somatic symptom burden and has demonstrated good psychometric properties across cultural settings, including its sensitivity to symptom variation and clinical relevance. Although formal cultural validation in the Pakistani context is limited, prior research underscores its utility as an effective screening tool for somatization symptoms within primary care and psychiatric populations, facilitating comparison with international studies. Its brevity and ease of administration also support its use in large epidemiological studies where somatic complaints are prevalent and often comorbid with depressive disorders^[10]^.

The PHQ-15 was administered in its Urdu-translated version, previously used in Pakistani clinical settings. The translation followed forward–backward methodology, and minor linguistic adjustments were made for cultural relevance. Interviews were administered by psychiatry residents trained in structured interviewing. Inter-rater reliability was assessed through paired evaluations conducted at the beginning of data collection and every 4 weeks, yielding a Cohen’s kappa of 0.82 for PHQ-15 scoring.

### Data analysis

Data analysis was carried out using SPSS v.26. Quantitative variables like age, BMI, questionnaire score were presented as mean and standard deviation, and qualitative data like gender, education, residence, marital status, profession, SE status, treatment status of depression, presence or absence of somatic symptoms were reported as frequencies and percentages. Data was stratified for age, gender, BMI, education, residence, marital status, and profession. Post-stratification chi-square test was applied, taking a *P*-value of ≤0.05 as statistically significant.

### Ethical considerations

Ethical consent was obtained from the ethical board meeting of PIMS Islamabad. Participation in the study was voluntary, and participants had the right to withdraw at any point without any consequence. All data were anonymized, and participant identities were kept confidential. Data were stored securely and was only accessible to the research team.

## Results

Gender wise distribution of the patients revealed that 74 were males (30.5%) and 169 were females (69.5%). In terms of age distribution, the largest age group was 12–25 years (*n* = 131, 53.9%), followed by 26–35 years (*n* = 63, 25.9%). The majority of participants were unemployed (*n* = 150, 61.7%). Most resided in self-owned homes (*n* = 185, 76.1%), located in urban areas (*n* = 164, 67.5%). BMI distribution showed that 132 participants (54.3%) had normal BMI. For disease duration, 96 participants (39.5%) reported illness lasting more than 2 years, 85 (35.0%) reported 1–6 months, 40 (16.5%) reported 1–2 years, and 22 (9.1%) reported 6–12 months, as reported in Table 1 (Demographic Distribution).

**Table 1:** Demographic distribution

	Category	Frequency
Gender	Male	74 (30.5%)
	Female	169 (69.5%)
Age (years)	12–25	131 (53.9%)
	26–35	63 (25.9%)
	36–45	25 (10.3%)
	46–60	24 (9.9%)
Monthly income (Rupees)	>100K	17 (7%)
	51K–100K	25 (10.3%)
	31K–50K	28 (11.5%)
	Under 30K	23 (9.5%)
	Unemployed	150 (61.7%)
Marital status	Married	95 (39.1%)
	Single	140 (57.6%)
	Widowed	3 (1.2%)
	Divorced	5 (2.1%)
BMI (kg/m^2^)	Underweight (<18.5 kg/m^2^)	25 (10.3%)
	Normal weight (18.5 to 24.9 kg/m^2^)	132 (54.3%)
	Overweight (25.0 to 29.9 kg/m^2^)	61 (25.1%)
	Obese (30.0 and above kg/m^2^)	25 (10.3%)
Residence ownership	Self-owned	185 (76.1%)
	Rented	58 (23.9%)
Residential area	Rural	79 (32.5%)
	Urban	164 (67.5%)
Disease duration (months)	1–6 months	85 (35%)
	6–12 months	22 (9.1%)
	13–24 months	40 (16.5%)
	More than 24 months	96 (39.5%)
Education	Uneducated	40 (16.5%)
	Secondary education	61 (25.1%)
	Graduate level education	75 (30.9%)
	Higher education	67 (27.6%)

Among the somatic symptoms that bothered the patients a lot, low energy was most frequently reported by 144 patients (59.3%), followed by troubled sleeping by 117 patients (48.1%). Painful sexual intercourse was least commonly reported by 09 patients (3.7%) as reported in Table 2 (Somatic symptoms among study participants).

**Table 2:** Somatic symptoms among study participants (*n* = 243)

Somatic Symptoms	Symptoms severity		
	Not bothered at all	Bothered a little	Bothered a lot
Stomach pain	104 (42.8%)	76 (31.3%)	63 (25.9%)
Back pain	68 (28.0%)	93 (38.3%)	82 (33.7%)
Pain in arms, legs and joints	53 (21.8%)	95 (39.1%)	95 (39.1%)
Menstrual cramps (in women)	132 (54.3%)	57 (23.5%)	54 (22.2%)
Headache	42 (17.3%)	110 (45.3%)	91 (37.4%)
Chest pain	137 (56.4%)	65 (26.7%)	41 (16.9%)
Dizziness	99 (40.7%)	76 (31.3%)	68 (28.0%)
Fainting spells	143 (58.8%)	70 (28.8%)	30 (12.3%)
Racing heart	64 (26.3%)	84 (34.6%)	95 (39.1%)
Shortness of breath	93 (38.3%)	86 (35.4%)	64 (26.3%)
Painful sexual intercourse	202 (83.1%)	32 (13.2%)	09 (3.7%)
Constipation, loose bowels, diarrhea	104 (42.8%)	76 (31.3%)	63 (25.9%)
Nausea, gas, indigestion	95 (39.1%)	74 (30.5%)	74 (30.5%)
Low energy	22 (9.1%)	77 (31.7%)	144 (59.3%)
Trouble sleeping	46 (18.9%)	80 (32.9%)	117 (48.1%)

High PHQ-15 score was observed in 107 patients (44.0%), followed by medium score (*n* = 73, 30.0%), and minimal score was least commonly observed in 18 patients (7.4%), as shown in Table 3 (Somatic symptoms severity among study participants).

**Table 3:** Somatic symptoms severity among study participants (*n* = 243)

PHQ 15 severity score	Frequency	Percent
Minimal (score 0 to 4)	18	7.4
Low (score 5 to 9)	45	18.5
Medium (score 10 to 14)	73	30.0
High (score 15 to 30)	107	44.0
Total	243	100.0

High PHQ-15 score was recorded in 52 patients (39.7%), aged 12–25 years, 26 (41.3%) patients aged 26–35 years, 12 (48.0%) patients aged 36–45 years, and 17 (70.8%) patients aged 46–60 years, respectively. The p-value was 0.011, which is statistically significant. No other significant association was recorded, as shown in Table 4 (Subgroup analysis of degree of somatization with sociodemographic parameters).

**Table 4:** Subgroup analysis of degree of somatization with sociodemographic parameters (*n* = 243)

	Severity PHQ-15				Total	
	Minimal	Low	Medium	High		*P*-Value
Participant age (years)
12–25	13	32	34	52	131	0.011
	9.9%	24.4%	26.0%	39.7%	100.0%	
26–35	4	8	25	26	63	
	6.3%	12.7%	39.7%	41.3%	100.0%	
36–45	1	1	11	12	25	
	4.0%	4.0%	44.0%	48.0%	100.0%	
46–60	0	4	3	17	24	
	0.0%	16.7%	12.5%	70.8%	100.0%	
Participant gender
Male	7	18	26	23	74	0.057
	9.5%	24.3%	35.1%	31.1%	100.0%	
Female	11	27	47	84	169	
	6.5%	16.0%	27.8%	49.7%	100.0%	
Participant marital status
Married	6	9	30	50	95	0.100
	6.3%	9.5%	31.6%	52.6%	100.0%	
Single	12	36	40	52	140	
	8.6%	25.7%	28.6%	37.1%	100.0%	
Widowed	0	0	1	2	3	
	0.0%	0.0%	33.3%	66.7%	100.0%	
Divorced	0	0	2	3	5	
	0.0%	0.0%	40.0%	60.0%	100.0%	
Participant BMI
Underweight	2	2	10	11	25	0.223
	8.0%	8.0%	40.0%	44.0%	100.0%	
Normal	12	32	32	56	132	
	9.1%	24.2%	24.2%	42.4%	100.0%	
Overweight	2	8	24	27	61	
	3.3%	13.1%	39.3%	44.3%	100.0%	
Obese	2	3	7	13	25	
	8.0%	12.0%	28.0%	52.0%	100.0%	
Residential area
Rural	6	18	20	35	79	0.567
	7.6%	22.8%	25.3%	44.3%	100.0%	
Urban	12	27	53	72	164	
	7.3%	16.5%	32.3%	43.9%	100.0%	
Residence ownership
Self-owned	14	34	58	79	185	0.852
	7.6%	18.4%	31.4%	42.7%	100.0%	
Rented	4	11	15	28	58	
	6.9%	19.0%	25.9%	48.3%	100.0%	
Participant education
Uneducated	2	6	11	21	40	0.265
	5.0%	15.0%	27.5%	52.5%	100.0%	
Secondary	3	8	23	27	61	
	4.9%	13.1%	37.7%	44.3%	100.0%	
Graduate	6	19	15	35	75	
	8.0%	25.3%	20.0%	46.7%	100.0%	
Higher	7	12	24	24	67	
	10.4%	17.9%	35.8%	35.8%	100.0%	

Spearman’s rank correlation showed weak but statistically significant monotonic associations between PHQ-15 somatization severity and age (*ρ* = 0.194, *P* = 0.002), gender (*ρ* = 0.186, *P* = 0.004), and number of chronic illnesses (*ρ* = 0.183, *P* = 0.001). Marital status demonstrated a weak negative association (*ρ* = –0.153, *P* = 0.017). BMI, residence, home ownership, and education were not significantly correlated with somatization severity. This is shown in Table 5 (Spearman’s rank correlation analysis).

**Table 5:** Spearman’s rank correlation analysis

Variable	Correlation (*ρ*)	*P*-Value	Interpretation (vs higher depression)
Age (coded)	0.194	0.002	Weak Positive: A higher coded value for age is associated with a higher phq15_total score.
Gender (code)	0.186	0.004	Weak Positive: A higher coded value for gender is associated with a higher phq15_total score.
Chronic illnesses (Number of)	0.183	0.001	Weak Positive: A greater number of chronic illnesses is associated with higher depression scores.
Marital status (code)	−0.153	0.017	Weak Negative: A higher coded value for marital status is associated with a lower phq15_total score.
BMI (code)	0.058	0.368	Relationship is not statistically significant.
Residential area	0.046	0.471	Relationship is not statistically significant.
Residence ownership (code)	0.069	0.287	Relationship is not statistically significant.
Education (code)	−0.101	0.117	Relationship is not statistically significant.

A preliminary multinomial logistic regression model was constructed, but the overall model fit was non-significant, and pseudo-R^2^ values were unstable, suggesting over-parameterization relative to the sample size. Therefore, multivariable analysis was restricted to a parsimonious model including age, gender, and education. In this reduced model, age remained the only statistically significant variable associated with higher PHQ-15 severity (*P* < 0.01), while gender and education were not significant predictors.

## Discussion

Our study found a high prevalence of somatic symptoms among adult patients diagnosed with depression, with 26% reporting minimal and low, 30% reporting medium, and 44% exhibiting severe somatic symptom burden on the PHQ15 Scale. This is comparable to the findings of previous research conducted in Pakistan and internationally, thus cementing the integral role of somatization in the clinical presentation of depression^[2,4]^. Previous studies have found a prevalence of 40–70% in Pakistani cohorts, highlighting the rationale for early referral of such patients to psychiatric services, ensuring swift diagnosis and treatment. This could lead to mitigation of the high burden of somatic symptoms on health systems by reducing unnecessary investigations and decreasing the time from presentation to correct diagnosis^[10]^.

Large international epidemiological studies have estimated a prevalence of 40–90% of somatic symptoms disorder in clinical depression cohorts^[2]^. Cultural variations in the symptom expression are well documented, with European and east Asian populations, repeatedly, identifying fatigue, pain, and insomnia as the most prominent disturb disturbances^[11–13]^. Age, female, gender, low education, and urban residence have been identified as risk factors^[14,15]^ for somatic expression in multiple nations, although gender disparities appear to be less prominent in Asian contexts^[16,17]^. This might be able to explain the discrepancy in our study, emphasizing that both male and female patients in our cultural settings have a high expression of somatic symptoms.

Age showed the strongest correlation to the degree of somatization out of all the sociodemographic correlates. Older patients aged 46–60 years were the most likely to exhibit severe somatic symptoms. This pattern has been observed across both national and international studies in the past^[11,18,19]^. Aging is related to a higher burden of chronic physical illness, cognitive decline, social isolation, and change in illness behavior. All of these factors may be associated with greater somatic symptom burden, and complicate the clinical presentation of depression^[20]^. These findings highlight the need for age-sensitive screening practices for somatic symptoms in both primary care and mental healthcare services, especially in the context of a growing geriatric population in the country.

In contrast to past studies reporting a female predominance^[14,15]^, our study failed to find a statistically significant gender difference in somatic symptom severity. This difference may be due to differences in cultural factors that shape illness expression in Pakistan and limitations of the sample size. This finding is, however, consistent with Glise *et al*^[19]^. The sociocultural milieu modulates gender related differences in somatic symptom expression through many different factors, such as stigma, health literacy, and gender roles. More targeted research is needed to clarify this association and to optimize gender sensitive mental health care^[21]^. Previous studies from Pakistan found a high prevalence of somatization and considered it to be the predominant mode of expression of depression across diverse populations^[4,5]^. Sociopolitical unrest, stigma towards mental health, and deeply entrenched gender roles in Pakistan worsen these presentations^[22]^. Historically, the predominance of somatization in women has been a theme echoed in several studies, especially emphasizing fatigue, headache, and gastrointestinal symptoms as the major complaints^[23]^.

Contrary to Husain *et al*^[5]^, who reported higher rates of somatization in educated individuals who had strong social support, our study found higher severity of somatization in individuals who had lower education levels. This might be attributable to low health literacy, poor awareness about mental health, and limited access to healthcare services, which is commonly seen in Pakistan’s rural population more than in its urban population^[24]^. In addition, socioeconomic adversity, primarily measured as low income and unemployment, may act as a contextual contributor by worsening psychosocial stressors that are linked to depression and somatic complaints. This is more so a concern in resource-limited countries such as Pakistan^[25]^. Li *et al* also highlighted the socioeconomic implications of somatic symptoms, demonstrating that increased health seeking and illness behavior, unjustified investigations, and absenteeism from work significantly heighten healthcare costs^[7]^.

Marital status is another factor that has been found to significantly affect somatic symptom expression in past literature. In our study, 52.6% of married individuals reported high somatic symptom severity, which is consistent with previous South Asian literature that has found that marital and familial stress leads to heightened psychological distress, which eventually contributes to greater somatic symptoms^[26]^. Contrastingly, single or widowed individuals may experience social isolation, which leads to varying symptom manifestations, thus highlighting the complexity and nuance of the effect of social support on modulating somatic symptoms.

The strengths of our study include a substantial sample size across a broad age range, use of DSM-V criteria for the diagnosis of depression, and the use of a widely used somatic symptom tool (PHQ-15), which has demonstrated acceptable performance in South Asian clinical settings despite limited formal cultural validation in Pakistan. Detailed stratified analysis provided insights into critical sociodemographic factors affecting somatic symptom expression.

### Implications and recommendations

Fatigue and sleep disturbance were found to be the most commonly reported somatic symptoms. These are known correlates of prolonged diagnostic times and delayed treatment in depression. This is consistent with analytic evidence that identifies the symptoms as sensitive indicators of the severity of depression and as predictors of chronicity^[12,27]^. There is a two-way relationship between sleeplessness and depression, where each condition worsens the other. There is thus the need for integrated care that addresses these intertwined factors^[13,16]^.

## Conclusion

In conclusion, somatic symptoms constitute a prevalent and clinically relevant expression of depression among Pakistani patients. This expression is strongly associated with age, and weakly associated with gender and marital status. Gender differences are less pronounced, possibly due to cultural variation. These findings highlight the need for systematic somatic symptom screening integrated with a socio-demographically sensitive clinical approach. Enhancing primary care capacity for earlier identification of at-risk patients and their prompt referral to mental health services might contribute to timely diagnosis, earlier initiation of appropriate treatment, and improved outcomes. These might lead to better care for depressed individuals and decrease the burden of unnecessary investigations and longer treatment times on the health system.

### Limitations

The limitations of our study are a cross-sectional design, which restricts causal inference between sociodemographic factors and somatic symptoms. Our sample was recruited from a single center, which may limit its generalizability to other levels of care or a community population. Another limitation is that PHQ-15 assesses somatic symptom presence and severity, but does not fully encompass the cognitive and behavioral dimensions of somatic symptom severity.

Limitations of this study include the use of non-probability consecutive sampling, which may introduce selection bias as participants were recruited based on availability and willingness rather than random selection. This sampling approach restricts the generalizability of the findings to the broader population. Additionally, the broad age range of 12–60 years combines adolescents and adults, populations that may differ significantly in the manifestation and correlates of depression and somatic symptoms. Although age was recorded, stratified analyses by developmental stage were not conducted, which might have obscured age-specific patterns. Finally, while descriptive analyses are presented, multivariate analyses controlling for potential confounders such as age, gender, and socioeconomic status were limited, which constrains causal inference regarding the observed associations. Future studies employing probabilistic sampling, narrower age cohorts, and rigorous multivariate modeling are warranted to address these limitations.

The use of consecutive, non-probability sampling likely introduced selection bias, as patients attending a tertiary-care psychiatric outpatient department may have higher symptom severity and different sociodemographic characteristics than community or primary-care populations. Longitudinal Research might be needed to confirm causation between sociodemographic factors and somatic symptoms. Future research may also focus on how clinical course and treatment outcomes of depression are differentially affected by different levels of somatic symptoms severity.

## Data Availability

Data are available on the request from authors.
